# Pitfall of Universal Pre-Admission Screening for SARS-CoV-2 in a Low Prevalence Country

**DOI:** 10.3390/v13050804

**Published:** 2021-04-30

**Authors:** Jiwon Jung, Jinyeong Kim, Joon Seo Lim, Eun Ok Kim, Mi-Na Kim, Sung-Han Kim

**Affiliations:** 1 Department of Infectious Diseases, Asan Medical Center, University of Ulsan College of Medicine, Seoul 05505, Korea; trueblue27@naver.com (J.J.); wooyajy@gmail.com (J.K.); 2 Division of Infectious Disease, Department of Internal Medicine, Hanyang University Guri Hospital, Guri 11923, Korea; 3 Clinical Research Center, Asan Medical Center, University of Ulsan College of Medicine, Seoul 05505, Korea; joonseolim@gmail.com; 4 Office for Infection Control, Asan Medical Center, Seoul 05505, Korea; asankimeunok@gmail.com; 5 Department of Laboratory Medicine, Asan Medical Center, University of Ulsan College of Medicine, Seoul 05505, Korea; mnkim@amc.seoul.kr

**Keywords:** SARS-CoV-2, COVID-19, universal screening

## Abstract

It is unclear whether universal PCR screening for SARS-CoV-2 in asymptomatic individuals prior to admission is useful. From April to December 2020, the positive rate of universal pre-admission screening was 0.005% (4/76,521) in a tertiary care hospital in Korea. The positive rates were not different between the periods (period 1 (daily new patients of <1 per million inhabitants) vs. period 2 (1–8.3 per million inhabitants) vs. period 3 (10.3 to 20 per million inhabitants); *P* = 0.45). Universal pre-admission screening for SARS-CoV-2 had a lower positive rate than that of symptom-based screening (0.005% vs. 0.049% (53/109,257), *p* < 0.001). In addition, seven patients with negative pre-admission test results had subsequent positive PCR during hospitalization, and four patients had secondary transmission. Universal pre-admission PCR screening may not be practical in settings of low prevalence of COVID-19, and negative PCR results at admission should not serve as a basis for underestimating the risk of nosocomial spread from asymptomatic patients.

## 1. Introduction

Asymptomatic or pre-symptomatic SARS-CoV-2 infection is not uncommon [1], and asymptomatic and pre-symptomatic persons account for more than 40% of all SARS-CoV-2 infections [2,3]. In addition, recent studies found that transmission risk may be higher in the presymptomatic period than the symptomatic period [4,5]. Nosocomial transmission of SARS-CoV-2 is a considerable threat in hospitals and nursing homes. As such, many hospitals in South Korea have adopted a universal RT-PCR-based pre-admission screening policy for SARS-CoV-2, and since September 2020, the Korean government has begun reimbursing universal PCR screening with pooling tests against SARS-CoV-2 at hospital admission by national insurance coverage. It is unclear whether universal PCR screening for SARS-CoV-2 in asymptomatic individuals prior to admission is practical. We thus calculated the positive rate of pre-admission SARS-CoV-2 PCR screening in asymptomatic individuals according to the community-wide incidence of COVID-19. In addition, we compared the positive rate of pre-admission screening in asymptomatic individuals and that of symptom- or epidemiologic risk-based screening.

## 2. Methods

### 2.1. SARS-CoV-2 Screening Strategy

This study was performed at a tertiary care hospital in Seoul, South Korea, where the number of admissions per year is approximately 145,000. On 29 April 2020, our hospital implemented a universal pre-admission screening policy for SARS-CoV-2 using RT-PCR from nasopharyngeal swabs, which was applied to individuals (including children) without symptoms, epidemiologic risk factors, or links to recent outbreaks in the community or hospitals. If patients had a medical condition such as nasopharyngeal cancer or status of post-nasopharyngeal surgery, they performed a throat swab or sputum collection for RT-PCR. SARS-CoV-2 PCR tests for universal screening were performed by pooling five specimens, as described previously [6]. We performed RT-PCR using Allplex 2019-nCoV assay (Seegene, Seoul, South Korea) for all pooled specimens, and automated RNA extraction systems (STARlet (Seegene, Seoul, Korea)) were used. However, for patients with symptoms associated with COVID-19 [7], epidemiologic risk factors, or links with known COVID-19 outbreaks, PCR tests against SARS-CoV-2 were individually performed without pooling (Allplex 2019-nCoV assay or STANDARD M nCoV Real-time Detection (SD Biosensor, Suwon-si, South Korea)). In addition, the risk of transmission and mortality of SARS-CoV-2 infection in immunocompromised patients is high, and social distancing could not be maintained in the rehabilitation unit or closed psychiatric ward. Therefore, we performed universal follow-up testing (PCR by nasopharyngeal swab) for high-risk patients without symptoms, including immunocompromised patients (those with hematologic malignancy, hematopoietic stem cell transplant recipient, solid organ transplant recipient) hospitalized in the rehabilitation unit or closed psychiatric ward on hospital day 4 beginning on 21 November 2020. Whenever a case of COVID-19 was detected, we performed a thorough contact tracing. We reviewed the closed-circuit television (CCTV) footage to identify the contacts, which included patients, guardians, visitors, and healthcare workers (HCWs) who stayed at or visited the ward. All contacts were interviewed especially about wearing a mask, a facial shield or goggles, and gloves and categorized according to the nature of the activity during exposure, duration of exposure, and the personal protective equipment (PPE) worn at the time of exposure. HCWs and inpatients or guardians who came into contact underwent SARS-CoV-2 PCR testing and were monitored for COVID-19-related symptoms on a daily basis. We determined the index patient and the directionality of infection transmission based on the symptom onset and spatiotemporal relationship through intensive discussion with government epidemiologists. This study was approved by the Institutional Review Board of our hospital with a waiver of the requirement of patient consent (IRB no. 2020-1608).

### 2.2. Definition

Epidemiologic link was defined as close contact or history of visiting the venue or space where the outbreak or cluster occurred. We updated the epidemiologic link of a pre-admission questionnaire form daily from a press release from the Korean CDC. Regardless of underlying disease or the presence of explainable cause of the symptoms, we considered an individual symptomatic if they had any COVID-19-associated symptom (e.g., fever, chill, headache, cough, myalgia, sore throat, nasal stuffiness, hyposmia, hypogeusia, or dyspnea). A domestic case was defined as a confirmed case without travel history abroad within 14 days and no contact history of imported cases.

### 2.3. Statistical Analysis

In this study, the results of universal screening with pooling tests for asymptomatic patients without epidemiologic risk factors for SARS-CoV-2 infection were analyzed to evaluate the usefulness of this strategy. We divided the study period (week 18 to week 52 of the year 2020) into period 1 (week 18–week 32) when the number of new average daily domestic cases was below 50 (<1 per million inhabitants), period 2 (week 33–week 48) when the number of new average daily domestic cases ranged between 50 and 417 (1 to 8.3 per million inhabitants), and period 3 (week 49–week 52) when the number of new average daily domestic cases ranged between 514 to 1000 (10.3 to 20 per million inhabitants). To compare the positive rates between the periods, we used a chi-square test in MedCalc Statistical Software version 18.10.2 (MedCalc Software Bvba, Sotend, Belgium). In addition, we compared the positive rates between universal screening and symptom/epidemiologic risk-based screening using a chi-square test. *p* value < 0.05 was considered as statistically significant. We excluded the secondary and tertiary cases of nosocomial outbreak of our hospital in this analysis.

## 3. Results

During the whole study period (week 18 to week 52 of the year 2020), a total of 185,778 PCR tests for SARS-CoV-2 were performed including 76,521 (41%) pre-admission screening tests in asymptomatic patients without epidemiologic links. We performed universal follow-up tests in a total of 824 high-risk patients. In addition, 16,978 tests were performed for hospitalized patients with symptoms in the same period (median (interquartile range) days from admission to first reported symptom; 3 [1,2,3]). The positive rate of total test was 0.031% (58/185,778; 95% CI, 0.024–0.040%), the positive rate of universal pre-admission test was 0.005% (4/76,521; 95% CI, 0.002–0.013%), the positive rate of symptom-based screening was 0.049% (53/109,257; 95% CI, 0.037–0.063%), the positive rate of universal follow-up test in hospitalized patients was 0.121% (1/824; 95% CI, 0.021–0.684%), and the positive rate of symptom-based screening during hospitalization was 0.035% (6/16,978; 95% CI, 0.016–0.077%).

**Figure 1:**
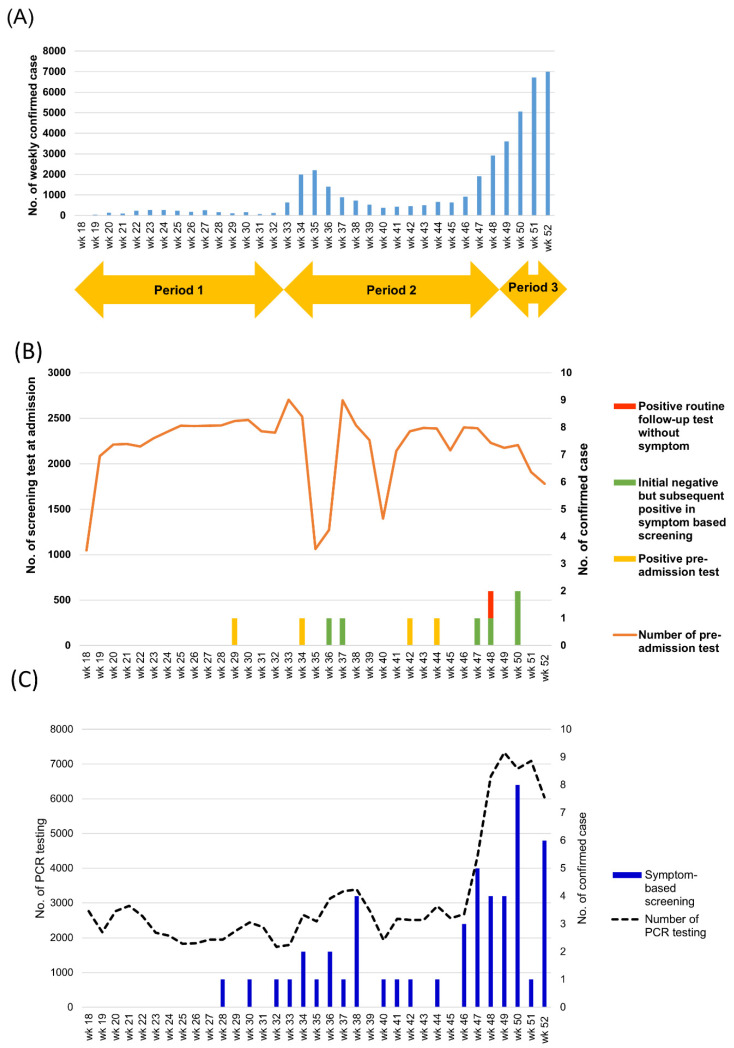
Trends of domestic cases of COVID-19 in South Korea and pre-admission screening tests at the Asan Medical Center. (**A**) Number of weekly domestic cases in South Korea between week 18 and week 52 of the year 2020. Period 1: new daily domestic cases < 1 per million inhabitants; period 2: new daily domestic cases: 1–8.3 per million inhabitants; period 3: new daily domestic case: 10.3–20 per million inhabitants. (**B**) Number of pre-admission screening tests (orange line) and the number of cases with positive results (yellow bars) and cases with initially negative results in pre-admission screening test, subsequent positive results in symptom-based screening (green bars), and universal follow-up testing (red bar) are shown. (**C**) Number of tests for patients with symptoms or epidemiologic link (dotted black line) and the number of cases with positive results are shown.

Of the 76,521 asymptomatic patients who underwent universal PCR screening, four (0.005%; 95% CI, 0.002–0.013%) patients revealed positive PCR results for SARS-CoV-2 (Figure 1). Regarding the detailed history of patients with a positive pre-admission test, one patient visited our emergency department for epigastric pain, nausea, and vomiting. She had a positive pre-admission test which was performed in emergency room. Another patient had positive pre-admission test which was performed one day before the planned admission. We found an epidemiologic link later that was unrecognized while a COVID-19 patient visited the restaurant where she worked. The remaining two patients initially reported no symptoms, but they had unrecognized minimal symptoms like dry cough and had no epidemiologic link.

The positive rates for SARS-CoV-2 in asymptomatic patients in period 1, period 2, and period 3 were 0.003% (1/33,687; 95% CI, 0.001–0.017%), 0.009% (3/34,768; 95% CI, 0.003–0.025%), and 0% (0/8966, 95% CI, 0–0.043%), respectively (*P* = 0.45 for 3 periods) (Figure 1). In contrast, symptom/epidemiologic risk-based screening during the whole study period had a significantly higher positive rate of 0.049% (53/109,257; 95% CI, 0.037–0.063%; *p* < 0.001 vs. universal screening). The positive rates for SARS-CoV-2 in symptomatic patients in period 1, period 2, and period 3 were 0.009% (3/33,730; 95% CI, 0.003–0.026%), 0.066% (29/44,188; 95% CI, 0.046–0.094%), and 0.08% (22/27,339, 95% CI, 0.053–0.122%), respectively (*P* = 0.0001 for 3 periods).

Notably, universal pre-admission screening during periods 2 and 3 missed six patients who showed positive PCR test results after developing symptoms during hospitalization, and one presymptomatic patient who showed a positive PCR test result for universal follow-up test on hospital day 4 (Figure 1, Table 1). Two patients had a positive PCR test which was done for evaluation of postoperative fever. When analyzing these seven patients, the median days from admission to diagnosis was five (range, 2–13), and four of seven patients had a secondary or tertiary transmission.

## 4. Discussion

Previous studies reported that the positive rates of universal pre-admission SARS-CoV-2 PCR screening were different according to the contemporary incidence of COVID-19 in the corresponding community. In New York City, as many as 29 (13.5%) of 215 pregnant women were revealed to be asymptomatic carriers of SARS-CoV-2 at admission between a 14-day period (22 March to 4 April 2020) when the number of new cases was rapidly rising [8]. During a similar period (2 March to 15 April 2020), however, Seattle had yet to experience a significant spike in the number of new cases, and universal screening in asymptomatic individuals revealed only one (0.6%) out of 170 individuals with a positive SARS-CoV-2 result while four (22%) of 18 symptomatic patients had positive results [9]. A recent study from New York City at the time of the post-peak phase of the outbreak reported that pre-operational screening revealed a positive rate of 0.3% [10]. However, we observed that universal pre-admission screening for asymptomatic patients showed no different positive rates according to the community prevalence. The reason for the low positive rate despite increasing community prevalence is unclear. Possible explanation is that universal screening for asymptomatic individuals might still reveal low positive rates in a very low prevalence country where the new average daily domestic cases were less than 20 per million like South Korea. Another explanation is that, as most universal-preadmission testing was performed in patients for planned admission, it is possible that patients awaiting admission were less likely to visit crowded places and tended to avoid high-risk behavior.

Significant higher positive rates in symptom- or epidemiologic risk-based screening than in pre-admission testing for asymptomatic patients was shown in our study. Similar to our finding, a Swiss study [11] showed that 8 of 2278 (0.4%) asymptomatic patients had positive universal admission screening tests, while 11.3% (60/529) of symptomatic patients had a positive test. The absence of COVID-19 symptoms or signs yielded a negative predictive value of 99.6% in the median daily incidence of 40 cases (IQR 27-87 cases) corresponding to a rate of 2.7 cases/100,000 inhabitants. Importantly, we found that universal pre-admission testing for SARS-CoV-2 in asymptomatic patients has a notable shortcoming in that due to the incubation period of SARS-CoV-2, negative test results at admission cannot assure that nosocomial infection would not occur. Therefore, it is important to thoroughly screen the in-patients for the development of symptoms and perform testing immediately. However, as shown in our study, one patient caused 10 cases of secondary and tertiary transmission during the pre-symptomatic period, suggesting that follow-up testing may be warranted in patients with high-risk factors (e.g., those with immunosuppression, hematologic malignancy [12], hospitalization in the rehabilitation unit [13], or history of stay in a long-term care facility [14]) or those who develop symptoms. Alternatively, given that we experienced nosocomial transmission despite the adoption of additional symptom-based testing after admission, universal routine follow-up PCR testing after universal pre-admission screening may be warranted in settings with a particularly high prevalence of COVID-19. Actually, we had one positive case for universal routine follow-up PCR testing. Similar to our experience, Klompas M. et al. reported nosocomial outbreak involving 15 patients and 42 healthcare workers despite universal pre-admission testing and infection control measures due to an incubation period [15]. They suggest universal follow-up testing three–four days after admission. We initially chose the follow-up PCR tests in selected patients due to the limited capacity of PCR testing in our hospital. High-risk patients were selected in terms of COVID-19-associated mortality (immunocompromised patients) and risk behaviors in a certain department (rehabilitation unit, psychiatric wards). However, we believe that universal follow-up screening might be helpful in a setting where the resource of PCR testing is plentiful. Furthermore, we selected the follow-up test at hospital day 4 given that median incubation period of COVID-19 was 5.1 days [16] and the median serial interval was 4.6 days [17]. However, a recent study revealed that twice weekly testing without self-isolation in a high-risk healthcare environment for asymptomatic individuals was effective for reducing the transmission from R_o_ 2.5 to less than R_o_ 1 [18]. Further studies are needed for the optimal candidates of asymptomatic screening and optimal follow-up interval.

It is worth noting that symptom-based testing during hospitalization detected a few cases (0.035% (6/16,978)), although this strategy showed a higher positive rate than universal screening for asymptomatic individuals. This finding indicates that a majority of admitted patients complained of COVID-19-associated symptoms, eventually resulting in negative SARS-CoV-2 PCR results at a low community prevalence of COVID-19. In addition, we experienced a nosocomial outbreak that was triggered by the patient who had negative PCR testing on admission but subsequent positive testing due to fever during hospitalization (Patient A in Table 1). This patient transmitted the virus during the presymptomatic period, so we performed universal follow-up testing (hospital day 4) beginning on 21 November 2020. A relatively small number of patients underwent universal follow-up testing during the study period. Therefore, cautious interpretation is needed on the usefulness of universal follow-up testing due to wide confidence intervals.

The usefulness of universal screening in the setting of new daily cases of more than 20 per million people could not be evaluated in our study; therefore, it remains to be determined whether universal screening is effective in settings with a high incidence of COVID-19, and further cost-effectiveness analysis is needed on the universal screening tests according to the incidence of COVID-19 in the community. Another limitation of this study included the single center setting and the absence of serologic tests against SARS-CoV-2 infection. In addition, some may argue that asymptomatic patients with positive PCR results against SARS-CoV-2 genes may detect patients who do not have a viable virus with lingering positive PCR results after acute COVID-19 mild symptomatic or asymptomatic infection.

In conclusion, compared to the positive rate of symptom-based screening (0.049% (53/109,257)), the low overall positivity rate (0.005% (4/76,521)) of universal pre-admission screening for SARS-CoV-2 at our hospital suggests that the prevalence of COVID-19 in the surrounding community should be taken into account when deciding the implementation of a universal screening policy. Furthermore, negative PCR results at pre-admission screening should not serve as a basis for underestimating the risk of nosocomial spread from asymptomatic patients.

## Figures and Tables

**Table 1:** Characteristics of patients who had negative pre-admission SARS-CoV-2 PCR testing but had a positive test during hospitalization or after discharge.

Patient	Epidemiologic Link	Symptom at Diagnosis	Days from Admission to Diagnosis	No. Secondary or Tertiary Transmission	Comment
A	No	Fever	5	10	Transmission occurred in a multi-patient room, shared shower room with poor ventilation, and utility room.
B	Exposure in the community 1 day before admission	Fever, cough	7 (from first admission)	0	Patient was exposed to SARS-CoV-2 on 2 September She was admitted on 3 September (negative SARS-CoV-2 PCR) then discharged on 4 September. Symptom developed on 7 September, and she was re-admitted because of premature labor and vaginal bleeding on 10 September (positive SARS-CoV-2 PCR).
C	Exposure from spouse	Fever 1 day after operation	2	0	-
D	No	None at diagnosis, Fever developed 2 days after diagnosis	4	3	Three secondary transmissions occurred in a multi-patient room.
E	No	Fever 1 day after operation	3	0	-
F	No	Myalgia	13	1	Patient was hospitalized from 24 November 2020 to 27 November 2020. She had negative PCR result on 23 November 2020, but had a positive PCR result on 7 December, which was performed by request of our infection control team for forward tracing of one nosocomial case. Transmission occurred in a multi-patient room.
G	Exposure from spouse	Fever	11	4	Caregiver also had a positive PCR, which was performed for contact tracing. It was not distinguishable whether secondary transmission originated from patient or caregiver. Transmission occurred in a multi-patient room (patients and caregivers) with a nurse.

## Data Availability

Not applicable.
